# Efficacy of Tuohy Needle in Oocytes Collection from Excised Mare Ovaries

**DOI:** 10.4061/2010/102591

**Published:** 2010-08-05

**Authors:** F. Cremonesi, K. Anderson, A. Lange-Consiglio

**Affiliations:** ^1^Università degli Studi di Milano, Large Animal Hospital, Reproduction Unit, via dell'Università 6, 26900 Lodi, Italy; ^2^University of Wisconsin-River Falls, River falls, WI 54022, USA

## Abstract

Two methods have been described to recover oocytes from equine follicles in excised ovaries: aspiration and scraping. Aim of this work was to develop an effective method for collecting equine oocytes using Tuohy needle and comparing this technique to aspiration and scraping, with or without tunica albuginea removal. This hollow hypodermic needle, usually employed for inserting epidural catheters, is designed with a slightly curved tip, shaped similar to a small curette. In unpeeled ovaries, the recovery rates of Tuohy needle group was higher (*P* < .05) than in the 16 g needle aspiration and in the scraped ovaries (57% versus 36% and 47%) while the rate of cumulus-intact oocytes was higher than aspiration (46.9% versus 39.36%) but lower than scraping (46.97%) (*P* < .001). In unpeeled ovaries there was significant difference in maturation rate of oocytes recovered by Tuohy needle in respect to peeled ovaries (58.54% versus 50.17%, resp.). Combination of aspiration and scraping by Tuohy needle allows a faster and reliable collection of oocytes suitable for horse IVM.

## 1. Introduction

Progress in *in vitro* maturation (IVM) of equine oocytes has been hampered by the difficulty in collecting oocytes in large enough numbers to perform meaningful experiments. Two methods have been described to recover oocytes from equine follicles in excised ovaries: aspiration and scraping. Aspiration has been found to give a low recovery rate (31–46%) [1, 2], and to yield oocytes largely denuded of cumulus. As an alternative, scraping of the granulosa layer results in a high recovery of cumulus-intact oocytes (71–85%) [2, 3]. The need to scrape the follicles derives from the tight connections between the cumulus and the membrana granulosa and between the latter and the follicle wall [4]. The collection of oocytes by scraping requires incision of follicles, scraping of the follicle wall with a curette and extensive flushing to detach the cumulus-oocyte complexes (COCs). This technique is reflected in an increased length of time and number of personnel required for collecting enough oocytes to carry out even the simplest experiment. Moreover, the too long holding time of oocytes delays the immediate in vitro culture for maturation of oocytes [5, 6]. It is obvious that all these difficulties represent a major limitation for basic research on equine oocytes and for all the downstream technologies from *in vitro *fertilization to ICSI, embryo culture, and cloning.

Aim of this work was to develop an effective method for collecting equine oocytes assembling the feature of aspiration (fastness) with that of scraping (high recovery rate of cumulus-intact oocytes). For this purpose a particular hypodermic needle, usually employed for inserting epidural catheters, known as Tuohy needle, was used. This hollow needle is designed with a slightly curved tip, shaped similar to a small curette. Furthermore, we compared cumulus morphology and maturation rates to metaphase II, among oocytes collected by this new technique and oocytes harvested by standard needle aspiration or scraping, with or without tunica albuginea removal from the ovary.

## 2. Materials and Methods

Ovaries were collected at a local abattoir, freed of any surrounding extra tissue and brought to the laboratory soon after slaughter in PBS supplemented with penicillin/streptomycin at 30°C. For all the experiments, part of the ovaries were used without removal of the tunica albuginea (unpeeling) or after peeling of the tunica to expose follicles. After voiding of all visible follicles, peeled or unpeeled ovaries were bisected along their midline at 10 mm intervals, from ovulation fossa to the greater curvature, and any newly exposed follicles were utilized.

All experiments were replicated three times and data were analyzed by one-way Analysis of Variance (ANOVA) using Student-Newman-Keuls Multiple Comparisons Test.

In the first experiment all follicles were opened with a scalpel blade and the granulosa cell layer was scraped with a curette following the method of Hinrichs et al. [7]. Granulosa cells were flushed from the curette into sterile 50-ml Falcon tubes which were maintained in waterbath at 38°C. After brief sedimentation, the pellets were collected using HEPES-buffered TCM tissue culture.

In the second experiment all follicles were aspirated using a 16-g (1.6 × 40 mm) needle (Becton Dikinson, NJ, USA) attached via silicone tubing to a Kmar 3000 vacuum pump (Cook, Australia) in turn connected to an embryo filter (EM-COM, Agtech, USA) maintained at 38°. The aspiration pressure was set at 70 mm Hg. During aspiration the needle was repeatedly rinsed with HEPES-buffered TCM medium. After collection the excess fluid was drained through the filter and the sediment poured into square petri dishes. 

**Figure 1:**
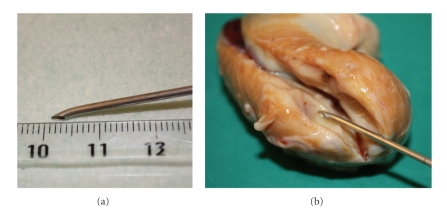
Tuohy needle showing its curved tip (a) and a representative image inside a sectioned 1 cm mare follicle (b).

In the third experiment all follicles were aspirated using a 16-g Tuohy needle (1.6 × 80 mm) (Epineed, Terumo Co., Japan) (Figures 1(a) and 1(b)) in the same conditions as above. During aspiration, a rotatory movement within the follicle helped dislodging the oocyte from the follicular wall.

In all experiments, collected cumulus-oocyte complexes (COCs) were classified as having compact, expanded (Ex), corona radiata only, or partial cumulus investments, depending on the presence and expansion of cumulus cells. Only Ex oocytes regardless of cytoplasmic appearance were used for this study, while all other COCs were used for other experiments.

The selected Ex oocytes collected throughout the experiments were matured in vitro (IVM) following the procedure by Dell'Aquila et al. [8]. After IVM, denuded oocytes showing 1st polar body extruded were classified as mature.

## 3. Results and Discussion

Mean number of follicles/ovary and oocytes/ovary, recovery rate and the cumulus morphology of oocytes collected in all experiments are reported, respectively, in Tables 1 and 2.

In the first experiment, 969 follicles were scraped with the curette from a total of 160 unpeeled ovaries with an average of 6.04 follicles per ovary. 973 follicles were scraped from a total of 143 peeled ovaries with an average of 6.80 follicles per ovary. To collect 100 oocytes, 4 people working for 4-5 hours with peeled ovaries and 3-4 hours with unpeeled ovaries were required.

In the second experiment 256 follicles were aspirated with the 16-g needle from a total of 41 unpeeled ovaries with an average of 6.24 follicles per ovary. 312 follicles were aspirated from a total of 38 peeled ovaries with an average of 8.21 follicles per ovary. To collect 100 oocytes, 3 people working for 2-3 hours with peeled ovaries and 1.5–2 hours with unpeeled ovaries were required.

In the third experiment 265 follicles were aspirated and scraped with the 16-g Tuohy needle from a total of 46 unpeeled ovaries with an average of 5.76 follicles per ovary. 1864 follicles were aspirated and scraped from a total of 273 peeled ovaries with an average of 6.83 follicles per ovary. There were no statistical differences in the mean number of oocytes per ovary when follicles were scraped or collected with Tuohy needle either comparing unpeeled ovaries or peeled ovaries (Table 1). In the Tuohy groups, the recovery rates, beside being significantly higher (*P* < .05) than in the 16-g needle aspiration groups and in the unpeeled scraped ovaries (Table 1), were yet significantly lower than in the group of ovaries peeled before scraping of follicles. Using Tuohy needle, the rate of cumulus-intact oocytes was higher than aspiration (46.9% versus 39.36%) but lower than scraping (46.97%) (*P* < .001) (Table 2). 

Table 3 shows the maturation rates of expanded cumulus oocytes collected by different techniques.

There were no differences in maturation rates of oocytes recovered in nonpeeled ovaries by Tuohy needle in respect to scraping in peeled ovaries.

Both collection techniques, classical aspiration, or scraping are widely employed on excised ovaries and the comparison between these two methods have been reported previously [2, 3].

In this study, we found that aspiration of follicles resulted in a lower oocyte recovery rate and higher prevalence of oocytes having partial cumulus than did follicle scraping which, in general, allow to recover oocytes with better quality. Similar results have been reported previously [1, 2], but this is the first report in which the two techniques were combined. Typically during scraping, follicles are opened and are no more suitable for aspiration. In this study, using Tuohy needle, scraping was performed on the same follicles while being aspirated.

In our study, the recovery rates obtained using the combination of aspiration and scraping by means of the Tuohy needle still remained significantly lower than that attained in peeled ovaries by scraping but maturation rates were not statistically different in these two groups. It is noteworthy that our technique improved the recovery rate of intact cumulus oocytes and maturation rate compared to aspiration by the conventional 16-g needle.

Moreover, the maturation rates of expanded cumulus oocytes collected by Tuohy needle in unpeeled ovaries and by scraping in peeled ovaries are similar. 

In conclusion, the collection technique used in this experiment assembling aspiration and scraping allow to recover intact-cumulus oocytes without altering the maturation rate, so this finding is very important because both peeling and scraping are time consuming and limiting factors for in vitro oocytes maturation in the horse.

## Figures and Tables

**Table 1:** Mean number of follicles and oocytes per ovary and recovery rate.

Collection technique	Follicles/ovary	Oocytes/ovary	recovery rate
			oocytes/follicles (%)
unpeeled and scraping	6.04 ± 2.27^a^	2.78 ± 0.94^a^	47 ± 0.02^a^
peeled and scraping	6.80 ± 0.20^b^	4.27 ± 0.30^b^	63 ± 0.03^b^
unpeeled 16-g needle	6.24 ± 0.85^ab^	2.29 ± 0.45^ab^	36 ± 0.08^c^
peeled 16-g needle	8.21 ± 0.68^c^	3.16 ± 0.34^c ^	39 ± 0.01^c^
unpeeled Tuohy needle	5.76 ± 0.30^a^	3.15 ± 0.76^ad^	57 ± 0.10^d^
peeled Tuohy needle	6.83 ± 1.15^b^	3.67 ± 0.77^be^	56 ± 0.12^d^

Values with different superscripts within the same column differ (*P* < .05).

**Table 2:** Cumulus morphology by different collection techniques.

Collection technique	no.	no.	Compact cumulus	Expanded cumulus	Partial cumulus
	ovaries	oocytes	no. (%)	no. (%)	no. (%)
unpeeled and scraping	160	445	209 (46.97 ± 0.13%)^a^	172 (38.65 ± 0.03%)^a^	64 (14.38 ± 0.03%)^a^
peeled and scraping	143	611	324 (53.02 ± 0.05%)^b^	170 (27.82 ± 0.08%)^b^	117 (19.15 ± 0.08%)^b^
unpeeled 16-g needle	41	94	37 (39.36 ± 0.01%)^c^	24 (25.53 ± 0.03%)^c^	33 ( 35.11 ± 0.03%)^c^
peeled 16-g needle	38	120	53 (44.17 ± 0.09%)^d^	16 (13.33 ± 0.03%)^d^	51 ( 42.5 ± 0.05%)^d^
unpeeled Tuohy needle	46	145	68 (46.90 ± 0.13%)^e^	41 (28.28 ± 0.09%)^e^	36 ( 24.83 ± 0.13%)^e^
peeled Tuohy needle	273	1003	491 (48.95 ± 0.05%)^f^	289 (28.81 ± 0.07%)^f^	223 (22.23 ± 0.09%)^f^

Values with different superscripts within the same column differ (*P* < .001).

**Table 3:** Maturation rates.

Collection technique	Ex oocytes no.	Maturation rate (no.) %
unpeeled and scraping	172	(108) 62.80 ± 2.15^a^
peeled and scraping	170	(99) 58.24 ± 1.36^b^
unpeeled 16-g needle	24	(11) 45.83 ± 3.61^c^
peleed 16-g needle	16	(7) 43.75 ± 1.55^d^
unpeeled Tuohy needle	41	(24) 58.54 ± 2.31^be^
peeled Tuohy needle	289	(45) 50.17 ± 2.52^f^

Values with different superscripts within the same column differ (*P* < .001).
